# What Do Teachers Think About Their Students’ Inclusion? Consistency of Students’ Self-Reports and Teacher Ratings

**DOI:** 10.3389/fpsyg.2019.01637

**Published:** 2019-07-16

**Authors:** Martin Venetz, Carmen L. A. Zurbriggen, Susanne Schwab

**Affiliations:** ^1^University of Applied Sciences of Special Needs Education, Zurich, Switzerland; ^2^Faculty of Educational Science, University of Bielefeld, Bielefeld, Germany; ^3^Department of Education, University of Vienna, Vienna, Austria; ^4^Research Focus Area Optentia, North-West University, Vanderbijlpark, South Africa

**Keywords:** self-report, teacher rating, assessment accuracy, inclusion, academic self-concept

## Abstract

The aim of this study was to investigate the consistency between the self-reports and teacher ratings of students’ emotional and social inclusion at school as well as for their academic self-concept. The German version of the Perceptions of Inclusion Questionnaire (PIQ) was administered to 329 grade 8 students (50.8% female, *M*_age_ = 14.5 years, *SD*_age_ = 0.5 years) and their teachers. First, the three-dimensional structure of both PIQ versions was confirmed by confirmatory item factor analysis. The α and ω coefficients demonstrated good reliability for all scales. Second, a correlated trait-correlated method minus one model provided evidence that the method-specificity of teacher ratings was larger than the consistency between the self-reports and teacher ratings. Third, the results of a latent difference model indicated that general method effects can partly be explained by a student’s gender or special educational needs. Finally, the low consistency between self-reports and teacher rating is discussed.

## Introduction

Inclusion is currently a hot topic in education. As such, responding to student diversity has brought about new challenges for teachers. Conceived in general terms, inclusive education represents a shift from a teaching approach that works for *most* students to one that involves the creation of learning opportunities for every student (For a brief history of the idea of inclusive education, see Armstrong et al., 2011). In order to meet diversity challenges in classroom, it is critical that teachers adapt their instructional practices. An important prerequisite for adaptive teaching is teachers’ diagnostic expertise, which is considered a central element of teacher professionalism (Praetorius et al., 2013; Artelt and Rausch, 2014).

Within an inclusive context, teachers are not only asked to enhance students’ learning outcomes, but also to support their social-emotional development. Therefore, a broader educational agenda in favor of positive social and emotional outcomes is often advocated (Prince and Hadwin, 2013). Educational systems, however, tend to focus on the acquisition of academic knowledge. Consequently, prior research in inclusive education often evaluated the academic outcomes of students, especially of those with special educational needs (SEN) with regard to learning. The majority of these studies indicated that students with SEN achieve higher academic achievement in inclusive educational settings than in special schools (meta-analysis of Oh-Young and Filler, 2015; review of Ruijs and Peetsma, 2009). For students without SEN, no differences in academic achievement were observed between inclusive and non-inclusive classes (review of Ruijs et al., 2010).

In particular, at the beginning of inclusive research studies, students’ academic self-concept was frequently investigated (meta-analysis of Bear et al., 2002; reviews of Chapman, 1988). The results demonstrated that students with SEN with regard to learning have a lower academic self-concept when attending an inclusive or regular school than when in a special school (e.g., Marsh et al., 2006). Already at that time, several authors referred to the significance of non-academic aspects of inclusion not least for students with SEN. According to the theoretically well-founded work by Haeberlin et al. (1989), in addition to the “performance-oriented inclusion” (i.e., academic self-concept), students’ social and emotional inclusion constituted the three fundamental pedagogical dimensions. In line with the broader agenda of inclusive education, more recent studies of the effects of inclusive education include social-emotional outcomes more frequently with a focus on social aspects (e.g., Krull et al., 2014; Oh-Young and Filler, 2015). However, previous studies indicate that students with SEN in particular are at risk of being less socially accepted by their classmates (Koster et al., 2010; Bossaert et al., 2013; Schwab, 2015; de Boer and Pijl, 2016). Findings regarding emotional inclusion are less common and less consistent. In general, studies of emotional inclusion – often referred to as emotional well-being – indicate that students with SEN are more prone to dislike the time they spend at school than students without SEN (McCoy and Banks, 2012; Skrzypiec et al., 2016). According to the findings of Zurbriggen et al. (2018), students with and without SEN do not differ in how they experience everyday school life.

Since students’ academic self-concept as well as their social and emotional inclusion are main outcome variables for inclusive education (Prince and Hadwin, 2013; DeVries et al., 2018), it is necessary that teachers identify students who are struggling in these aspects, in order to offer appropriate support or to intervene at an early stage. Moreover, a teacher’s ability to accurately assess a student’s characteristics is thought to facilitate adaptive teaching (Südkamp et al., 2014) and to affect each student’s personal and academic development (Machts et al., 2016). Therefore, the present study aims to explore the accuracy of teachers’ assessments of these three constructs.

### Accuracy of Teachers’ Assessments of Students’ Academic or Cognitive Characteristics

The accuracy of a teacher’s assessments is often referred to as the teacher’s judgment accuracy, diagnostic expertise, or diagnostic competence. Most studies of teachers’ assessment accuracy focus on students’ academic achievement or cognitive abilities. In general, achievement is one of the student characteristics that teachers assess relatively well. According to the meta-analysis of Südkamp et al. (2012), which included 75 studies, teacher ratings of students’ *academic achievement* and students’ performance on a standardized achievement test were on average highly correlated (*r* = 0.63). Nevertheless, the range of these correlations indicated considerable heterogeneity in the primary study effects. These results were similar to an older review by Hoge and Coladarci (1989). The teachers’ assessment accuracy of students’ *cognitive abilities* is lower than the accuracy of academic achievement. The meta-analysis of Machts et al. (2016), which was based on 33 studies, reported a moderate mean correlation (*r* = 0.43) between test results and teachers’ assessments of students’ cognitive abilities. However, the results also revealed substantial variation in the effect sizes across studies.

Compared to research on teachers’ assessment accuracy with regard to students’ academic achievement or cognitive abilities, there are fewer studies of students’ *academic self-concept* (i.e., a student’s cognitive representations of his or her academic abilities). Most of these studies showed that a student’s self-concept is difficult to assess by a teacher. Urhahne et al. (2011), for example, found moderate correlations between the teachers’ assessments of students’ academic self-concept and students’ self-reports (*r* = 0.43). Praetorius et al. (2013) reported relatively low overlap for the self-concept in German as the language of instruction (*r* = 0.22) and for the self-concept in mathematics (*r* = 0.27). In the study by Zhu and Urhahne (2014), in turn, the teachers’ assessment accuracy with regard to students’ academic self-concept was high, independently of whether single-item (*r* = 0.65) or multiple-item measurements (*r* = 0.67) were used.

The considerable heterogeneity in the study effects indicates that teachers’ assessment accuracy may be biased. In the heuristic model of teacher judgment accuracy proposed by Südkamp et al. (2014), several teacher, student, judgment, and test characteristics are thought to influence the correspondence between teachers’ assessments of students’ academic achievement and the students’ actual test performance. In the context of inclusive education, the students’ SEN status is of particular interest. Furthermore, students’ gender and age are variables that are commonly taken into account in educational research.

The results of Hurwitz et al. (2007) revealed, for example, less accurate assessments in mathematics achievement for students with SEN (primarily with regard to learning) compared to students without SEN. In support of these results, Meissel et al. (2017) reported systematically lower teachers’ assessments of students’ reading and writing achievement for students with SEN than for students without SEN. Also, female students typically received higher teacher assessments than males. Moreover, Sommer et al. (2008) pointed out that teachers more frequently attribute higher cognitive abilities to female students than to male students. Regarding the academic self-concept, Praetorius et al. (2017) found higher teacher assessments for girls than for boys, in addition to higher assessments for younger students as well as for students with a very low or very high grade point average (GPA). However, students’ gender and age were not related to teachers’ assessment accuracy of students’ academic self-concept, whereas students with low or high GPAs were assessed more accurately than students with an average GPA.

### Accuracy of Teachers’ Assessments of Students’ Social and Emotional Inclusion

Teachers’ assessment accuracy with respect to students’ *social inclusion* has been largely neglected in previous research. Most studies have mainly focused on the social position of students. Social position is typically assessed by means of sociometric questionnaires (i.e., peer nomination), thus representing classmates’ views of a student’s social status (e.g., popularity) in class and not the student’s own view. Kwon et al. (2012), for example, found moderate correlations between the number of peer nominations and teachers’ ratings of students’ popularity (*r* = 0.42). van Ophuysen (2009), on the other hand, investigated the teachers’ assessment accuracy with regard to students’ peer relationships in class. Her results showed no significant correlations between teacher ratings and student ratings.

Gomez (2014) compared self-reports with teachers’ ratings of students’ emotional and behavioral problems in school, which were assessed using the widely used Strength and Difficulties Questionnaire (SDQ; Goodman, 2001). There was support for convergent validity between students’ and teachers’ ratings, especially for the scales emotional symptoms and peer problems. The low consistency for the other three scales was explained by the fact that these behaviors (e.g., conduct problems, i.e., student lies, cheats, or steals) are less observable for teachers than emotional symptoms or peer problems.

Teachers’ assessments of students’ *emotional inclusion* – i.e., emotional well-being at school – has received little attention in previous studies. Due to its internal characteristics, assessing students’ emotional well-being is even more difficult for teachers than other aspects because they need to infer students’ emotions (see the realistic accuracy model of Funder, 1995, 2012). Accordingly, Givvin et al. (2001) detected only low correlations between teachers’ ratings and self-reports of students’ positive and negative emotions during mathematics lessons. Similar results were reported in the study of Karing et al. (2015). They concluded that teachers did not accurately assess students’ emotionality and worries in the subjects German and mathematics. In the study of Zhu and Urhahne (2014), moderate agreement was found for teachers’ assessments of students’ learning enjoyment, whereas the agreement concerning students’ text anxiety was around zero. In another study, Urhahne and Zhu (2015) found that teachers could capture positive aspects of students’ subjective well-being with higher accuracy than negative ones. Nevertheless, teachers assessed students’ subjective well-being at school only with low to moderate accuracy.

Because of the relatively low overall level of agreement regarding social and emotional inclusion or well-being, researchers have examined the external variables that explain the overlap between students’ and teachers’ assessments. The results of Koster et al. (2007) indicated that teachers systematically overestimated the social position of students with SEN. In the study by de Monchy et al. (2004), about half of the teachers rated the social position of students with SEN too high. Sommer et al. (2008) examined the possibility of gender bias influencing teachers’ assessments of students’ social competencies. However, social competencies of boys were not easier to assess for teachers than those of girls. Urhahne and Zhu (2015) reported a small gender bias regarding students’ subjective well-being at school as indicated by higher teachers’ ratings for positive attitudes toward school, psychical complaints, and worries for girls compared to boys. Nonetheless, the interaction term (referring to teachers’ assessment accuracy) was only significant for physical complaints. Moreover, there was a bias concerning students’ achievement insofar that teachers’ ratings of students’ positive attitudes toward school, enjoyment, and, in particular, of their worries differed more between high and low achievers than the students’ ratings.

Taken together, teachers’ assessment accuracy of students’ self-concept can be considered moderate. The accuracy of social and emotional inclusion is rather low. The low to moderate convergent validity for these three constructs suggests an assessment bias (i.e., method effects). Students’ gender, academic achievement (or GPA), and, in particular, the SEN status seem to influence teachers’ assessment accuracy.

### The Perceptions of Inclusion Questionnaire

To the best of our knowledge, only one instrument is currently used to assess students’ emotional inclusion (i.e., emotional well-being in school), social inclusion (i.e., social relationships with other students), as well as the academic self-concept from the students’ and teachers’ perspectives. This tool is the Perception of Inclusion Questionnaire (PIQ; Venetz et al., 2015). The PIQ is a short questionnaire composed of three scales with four items each on a 4-point Likert scale. It is based on the German self-report Questionnaire for Assessing Dimensions of Integration of Students (FDI 4–6; Haeberlin et al., 1989). The PIQ is designed for students from grade 3 to 9 and can be self-administered or completed by the student’s teacher or by his or her parents or (primary) caregivers. The three versions of the PIQ are available online in German, English, and several other languages ^1^.

The development of the highly reliable short questionnaire by Venetz et al. (2014) included an initial examination of the construct validity of the scales. Evidence for convergent validity for the scale emotional inclusion was provided by a high positive correlation with affective states during lessons (*r* = 0.55), for the scale social inclusion by moderate negative correlations with teachers’ reports of student’s problems with peers (*r* = –0.45), and for the scale academic self-concept by high correlation with another self-concept scale (*r* = 0.72), in addition to moderate positive correlations with academic achievement in mathematics and German (*r* = 0.46 and 0.40, respectively). Divergent validity for the scale social inclusion, for example, was supported by a low correlation with the teacher’s report of students’ emotional problems (for more information, see Venetz et al., 2014; and Electronic Supplementary Material provided by Zurbriggen et al., 2017).

In the study by Zurbriggen et al. (2017), the PIQ student version (PIQ-S) was further evaluated by means of the multi-unidimensional graded response model (GRM). Initial analyses by means of exploratory and confirmatory factor analyses supported that the PIQ-S correspond with a multi-unidimensional three-factor structure, meaning that the three factors represent discrete latent dimensions measuring a common underlying construct. The results of the GRM analyses indicated that the scales meet high psychometric standards. As expected, the items provided considerable information at average or below-average levels of the three constructs emotional inclusion, social inclusion, and academic self-concept. Furthermore, nested logistic regression showed that differential item functioning for students with learning difficulties versus without learning difficulties was either absent or negligible, indicating that items measure equivalently across both groups. Comparisons between those groups are thus allowed.

Further empirical evidence comes from a recent study by DeVries et al. (2018), which demonstrated that the three scales of the PIQ-S possess strong measurement invariance across grade level (6th and 7th grade), gender, and for students with SEN versus without SEN. The PIQ-S is applicable for students with SEN. Their findings also showed support for content validity of the PIQ-S because the three scales correlated with the SDQ subscales in the expected directions.

Although previous research indicates that the psychometric properties of the PIQ-S are high (Venetz et al., 2014; Zurbriggen et al., 2017; DeVries et al., 2018), those of the PIQ teacher version (PIQ-T) have not yet been tested. In particular, no studies have investigated the convergent validity of both PIQ versions.

### The Present Study

Against this backdrop, the present study aims to examine teachers’ assessment accuracy of students’ internal perspectives of their inclusion at school. To be more precise, we investigated the consistency between self-reports and teacher ratings of students’ emotional and social inclusion as well as their academic self-concept using the PIQ-S and the PIQ-T. Because the PIQ-T has not been evaluated to date, we first tested its psychometric properties. Specifically, we tested the postulated three-factor structure of the PIQ and evaluated the reliability of the three subscales emotional inclusion, social inclusion, and academic self-concept. Second, we examined the consistency between self-reports and teacher ratings as well as method effects (i.e., method bias or method specificity) of the teacher ratings. Finally, we investigated whether the three selected student characteristics (gender, age, and SEN) can predict the method effects of teacher reports.

## Materials and Methods

### Sample

The data originated from the Austrian longitudinal study ATIS-SI (Attitudes Toward Inclusion of Students with Disabilities Related to Social Inclusion), which included primary and secondary school students from inclusive classes. The research was approved by the Styrian Regional School Authority.

In the present study, we used data from the 3rd measurement wave, which took place from May to June 2015 and had students from grade 8 participating in a paper-and-pencil survey. Informed consent was obtained from students and their parents. Furthermore, the class’s teacher completed a questionnaire about every student taking part in the study.

The sample consisted of 329 students (50.8% female, *M*_age_ = 14.5 years, *SD*_age_ = 0.5 years) from 20 classes. Thirty-five students (10.6%) were diagnosed as having SEN. In Austria, students with SEN need an official status recognized by the local educational authority in order to be eligible for additional resources. Thus, teachers were asked to list all children in their class officially acknowledged as having SEN. In Austria, students with SEN with regard to learning are described as students with a lower intelligence and lower academic competences compared to their peers. They exhibit general learning difficulties that are not just related to dyslexia or dyscalculia (Gebhardt et al., 2013).

The consent obtained from the teachers and the parents of the participants was both written as well as informed. The ethic committee of the regional school board of Styria, which is the local school authority of the part of Austria where the project took place, gave the ethical approval for the present study. An ethics approval by an institutional review board was not required as per applicable institutional and national guidelines and regulations.

### Measures

To assess students’ emotional inclusion, social inclusion, and academic self-concept, both students and teachers were asked to fill out the respective versions of the PIQ. The three PIQ subscales emotional inclusion (e.g., “I like going to school”), social inclusion (e.g., “I have very good relationships with my classmates”), and academic self-concept (e.g., “I do well in my schoolwork”) consist of four items each. The Likert-type items have four response categories: 0 = *not at all true*, 1 = *rather not true*, 2 = *somewhat true*, and 3 = *certainly true*. Students needed approximately 5 min to fill out the 12 items of the PIQ-S. The items of the PIQ-T were slightly modified in wording, but not in meaning (e.g., item #2, student’s version: “I am a fast learner,” teacher’s version: “He/she is a fast learner”).

### Analyses

First, categorical confirmatory item factor analyses (CCFA) were fitted and compared to assess the dimensional structure of both PIQ versions; particularly, that of the teacher’s version. To assess the fit of models, we used the chi-square test as well as sample size-independent goodness-of-fit indices: the comparative fit index (CFI), the Tucker-Lewis index (TLI), and the root mean square error of approximation (RMSEA). TLI and CFI values greater than 0.95 indicate a good fit to the data. RMSEA values less than 0.06 reflect close fit to the data (Hu and Bentler, 1999). To evaluate the reliability of the subscales, Cronbach’s α was estimated. Given the limitations of Cronbach’s α (Dunn et al., 2014), McDonald’s ω was calculated, in addition in the CCFA framework. According to Sijtsma (2009), Cronbach’s α corresponds in many cases to a lower bound of reliability.

**FIGURE 1:**
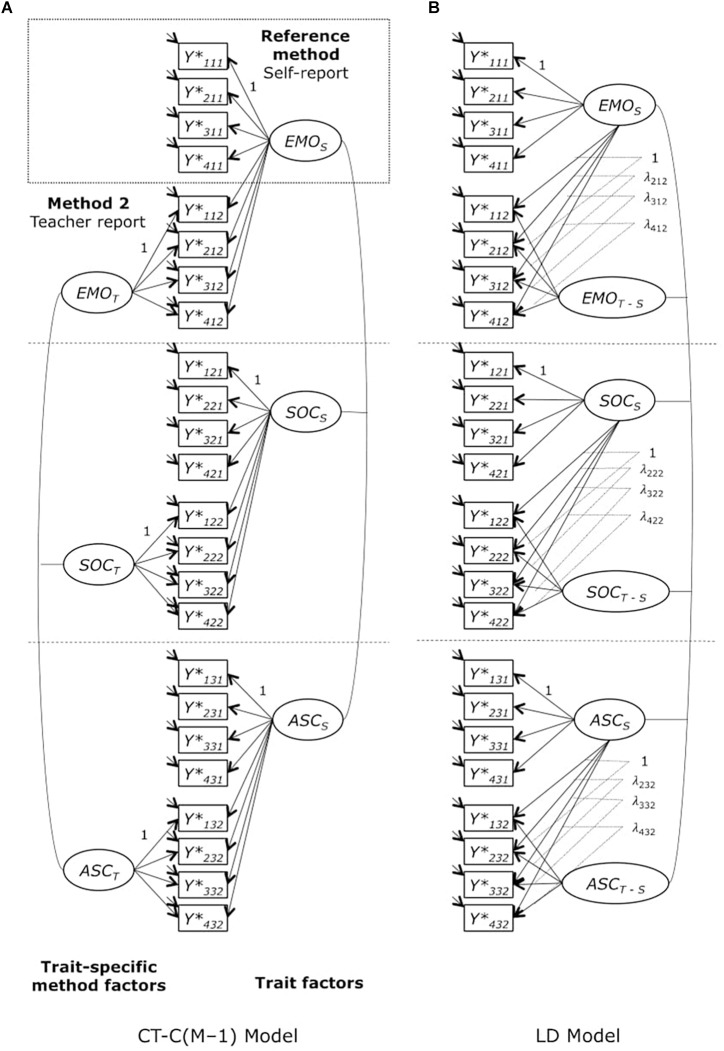
**(A)** Multiple-indicator correlated trait-correlated method minus one [CT-C(M–1)] model for ordinal variables, **(B)** Latent difference (LD) model for ordinal variables. *Y*^∗^_ijk_ = observed variable (*i* = indicator, *j* = trait, *k* = method); *EMO*_S_, *SOC*_S_, *ASC*_S_ = latent trait factors measured by self-report (reference method); *EMO*_T_, *SOC*_T_, *ASC*_T_ = latent trait-specific method factors (teacher report); *EMO*_T-S_, *SOC*_T-S_, *ASC*_T-S_ = latent difference method factors (difference between teacher report and self-report); λ_ijk_ = factor loadings. *EMO*, emotional inclusion; *SOC*, social inclusion; *ASC*, academic self-concept.

Second, we applied a correlated trait-correlated method minus one [CT-C(M–1)] model (Eid, 2000; Eid et al., 2003) to examine the convergent validity of self-reports and teacher ratings. The CT-C(M–1) model is a prominent confirmatory factor analysis multi-trait-multi-method (CFA-MTMM) approach for structurally different methods in which a method effect is defined relative to another method and is a latent residual variable (Eid et al., 2016). In the CT-C(M–1) model, the part of a trait measured by a specific method that cannot be predicted by the standard or reference method corresponds to the method effect (i.e., the specificity of the nonreference method). In this study, we selected student’s self-report as the reference method, as we considered the internal perspective to be the crucial perspective to assess students’ inclusion in school. The teacher report represented the non-reference method that was contrasted against the self-report (Figure 1A). One of the advantages of the CT-C(M–1) model is the possibility of estimating variance coefficients of consistency and method specificity. The consistency coefficient serves as an indicator of convergent validity relative to the reference method, whereas the method specificity coefficient represents the proportion of observed variance that is not shared with the reference method (Geiser et al., 2014). Nonetheless, in the CT-C(M–1) model, it is not possible to relate explanatory variables to the method factor if one of the variables is correlated with the trait factor because this will lead to a suppression structure (Koch et al., 2017).

Another CFA-MTMM model for structurally different methods is the latent difference model (LD; Pohl et al., 2008). Both models, the CT-C(M–1) model and the LD model, fit the data equally well (Geiser et al., 2012). As an aside, Geiser et al. (2012) showed that the restricted CT-C(M–1) model and the LD model represent direct reformulations of the MTMM correlation model proposed by Marsh and Hocevar (1988). In the LD model, however, method effects are defined as latent difference scores (i.e., the difference between the true score variable of the non-reference method and the true score variable of the reference method), representing a general method effect. This allows to directly relate explanatory variables to the method factors (Geiser et al., 2012).

Thus, third, we also applied an LD model to explain general method effects (Figure 1B). We included students’ gender, age, and SEN simultaneously as explanatory variables into the model. In contrast to the CT-C(M–1) model, a prerequisite of the LD model is that the structurally different methods share a common metric (Geiser et al., 2012, 2014). Therefore, we had to establish strong measurement invariance in advance to ensure that the method effect could be meaningfully interpreted as the difference between the two true-score variables corresponding to different methods (i.e., self-reports and teacher reports). To compare the nested models when examining measurement invariance, we performed chi-square difference tests. Since this test is known to be very sensitive to sample size (e.g., Marsh et al., 1988), we also considered the change in fit indices. It has been suggested that the more restrictive model is preferred if the change is not significantly worse than those of the less restrictive model. For CFI and TLI, the change should be less than 0.01, and for RMSEA, it should be less than 0.015 (Chen, 2007).

All analyses were carried out using M*plus* Version 8.0 (Muthén, 1998–2017). In accordance with the recommendations based on simulation studies (Nussbeck et al., 2006; Rhemtulla et al., 2012), parameters were estimated using the weighted least square means and variances (WLSMV) estimator.

Given the non-independence of observations (i.e., every teacher rated the students from his or her class), we used the complex sample option. In this design-based approach, the standard errors are adjusted according to the clustered data structure. The intraclass correlation coefficients for the 12 PIQ items from the teacher ratings ranged from 0.01 to 0.20 (*M* = 0.13).

## Results

### Descriptive Statistics and Factor Structure of the PIQ-S and the PIQ-T

Table 1 presents the distribution of responses, the mean, and the standard deviation for the items of the PIQ-S and the PIQ-T. Reverse coding was used for the negatively formulated items (i.e., items 4, 8, and 12). As expected, the item difficulties were found to be low. Modal values were in the highest or second highest category. Comparisons of response frequencies showed that the lowest category of the scale emotional inclusion was chosen more infrequently by teachers than by students (e.g., item 7: 14.9% vs. 2.4%), while the lowest category of the scale academic self-concept was rarely chosen by students (e.g., item 9: 4.0% vs. 15.8%). There was minimal missing data: between 0 and 0.6% per item for student ratings, and between 0.6 and 2.7% for teacher ratings.

**Table 1:** Item statistics for the PIQ student (PIQ-S) and the PIQ teacher version (PIQ-T).

		PIQ-S							PIQ-T						
			
				Category (%)							Category (%)				
							
No.	Item (student version)	*M*	*SD*	1	2	3	4	*MV*	*M*	*SD*	1	2	3	4	*MV*
	**Emotional inclusion** (α_PIQ-S_ = 0.86; α_PIQ-T_ = 0.92)	**10.49**	**3.18**						**11.83**	**2.81**					
1	I like going to school.	2.64	0.93	13.1	28.3	40.1	18.2	0.3	2.99	0.74	3.0	17.9	54.7	23.4	0.9
4	I have no desire to go to school. (–)	2.66	0.97	15.2	24.0	39.8	20.7	0.3	2.98	0.88	5.5	22.2	38.9	31.0	2.4
7	I like it in school.	2.67	0.94	14.9	21.3	45.0	18.2	0.6	2.99	0.72	2.4	18.5	54.1	22.8	2.1
10	School is fun.	2.52	0.91	15.2	30.7	40.1	13.7	0.3	2.87	0.75	3.3	24.3	51.4	18.2	2.7
	**Social inclusion** (α_PIQ-S_ = 0.76; α_PIQ-T_ = 0.86)	**13.85**	**2.01**						**12.67**	**2.55**					
2	I have a lot of friends in my class.	3.34	0.68	1.2	8.5	45.6	44.4	0.3	2.95	0.80	2.5	26.4	43.8	26.7	0.6
5	I get along very well with my classmates.	3.43	0.64	0.9	5.5	42.9	50.5	0.3	3.19	0.70	1.8	11.2	52.6	33.7	0.6
8	I feel alone in my class. (–)	3.66	0.66	2.4	3.3	20.1	73.9	0.3	3.35	0.83	4.3	10.0	31.3	53.2	1.2
11	I have very good relationships with my classmates.	3.43	0.65	0.9	6.1	42.2	50.5	0.3	3.19	0.69	1.2	12.2	51.7	33.1	1.8
	**Academic self-concept** (α_PIQ-S_ = 0.78; α_PIQ-T_ = 0.95)	**11.65**	**2.47**						**10.64**	**3.62**					
3	I am a fast learner.	2.83	0.85	5.8	28.6	42.6	22.8	0.3	2.61	0.91	12.8	29.8	39.8	16.7	0.9
6	I am able to solve very difficult exercises.	2.72	0.82	8.2	26.4	49.8	14.9	0.6	2.60	0.99	15.2	31.3	31.0	21.9	0.6
9	I do well in my schoolwork.	2.93	0.77	4.0	21.6	52.3	22.2	0.0	2.62	0.99	15.8	27.1	34.7	21.3	1.2
12	Many things in school are too difficult for me. (–)	3.19	0.74	3.0	10.3	51.1	35.0	0.6	2.82	1.01	11.2	27.3	28.9	31.3	2.1


*N = 329. (–), reversely coded items. 1 = *not at all true*, 2 = *somewhat not true*, 3 = *somewhat true*, 4 = *certainly true*; MV, missing values. Mean and standard deviation of the scales (in bold) rely on sum scores.*

Table 1 also shows the scale statistics. The scale mean for emotional inclusion was somewhat lower in self-reports than in teacher reports (10.49 vs. 11.83), whereas the self-reports were somewhat higher for social inclusion (13.85 vs. 12.67) and academic self-concept (11.65 vs. 10.64). Furthermore, the internal consistencies were acceptable to high. For the PIQ-T, Cronbach’s α was higher (0.86 ≤ α ≤ 0.95) than for the PIQ-S (0.76 ≤ α ≤ 0.86).

For both PIQ versions, a three-factor CCFA model was estimated. Both models showed an appropriate fit to the data as follows: PIQ-S (Table 2, Model 1a): χ^2^_WLSMV_ (51, *N* = 329) = 78.44, *p* = 0.008, CFI = 0.988, TLI = 0.985, RMSEA = 0.040, *p*_RMSEA_ = 0.809; PIQ-T: χ^2^_WLSMV_ (51, *N* = 329) = 85.75, *p* = 0.002, CFI = 0.997, TLI = 0.987, RMSEA = 0.046, *p*_RMSEA_ = 0.654 (Table 2, Model 1b).

**Table 2:** Summary of goodness-of-fit statistics for the CCFA, CT-C(M-1), and LD models.

Model	χ^2^_Wlsmv_	*df*	*p*	CFI	TLI	RMSEA	*p*_Rmsea_	Δχ^2^	Δ*df*	*p*
**(1) CCFA models**										
(1a) PIQ-S	78.44	51	0.008	0.988	0.985	0.040	0.809			
(1b) PIQ-T	85.75	51	0.002	0.997	0.997	0.046	>0.999			
**(2) CT-C(M–1) models**										
(2a) Baseline model	304.23	228	<0.001	0.992	0.991	0.032	>0.999			
(2b) Model with NWF	299.33	226	<0.001	0.993	0.991	0.031	>0.999	9.34	2	0.009
**(3) LD models**										
(3a) Configural invariance	304.23	228	<0.001	0.992	0.991	0.032	>0.999			
(3b) Weak invariance	314.22	237	<0.001	0.992	0.991	0.031	>0.999	16.44	9	0.058
(3c) Strong invariance	339.94	258	<0.001	0.992	0.991	0.031	>0.999	39.32	21	0.009
(3d) Model with explanatory variables	384.82	314	0.004	0.993	0.992	0.026	>0.999			


*CT-C(M–1), correlated trait-correlated method minus one; LD, latent difference; CFI, comparative fit index; TLI, Tucker–Lewis index; RMSEA, root mean square error of approximation. Δχ^*2*^, Chi-square difference tests (with reference to the previous model).*

In addition to Cronbach’s α, ω coefficients (McDonald, 1999) were calculated in the CCFA framework. Similar to the α coefficients, the ω coefficients for the three scales of the PIQ-T were higher than for the PIQ-S (e.g., emotional inclusion: ω = 0.96 vs. 0.91; Table 3). Furthermore, the PIQ factors showed moderate to high correlations. The average correlation of the teacher version with *M* = 0.61 was significantly higher than that of the student version with *M* = 0.40 (*z* = -3.64, *p* < 0.001).

**Table 3:** Correlations, means, variances, and reliabilities (ω) of the PIQ factors.

	1 PIQ-S/PIQ-T	2 PIQ-S/PIQ-T	3 PIQ-S/PIQ-T
(1) Emotional inclusion	0.91/0.96		
(2) Social inclusion	0.42^∗∗∗^/0.67^∗∗∗^	0.85/0.91	
(3) Academic self-concept	0.50^∗∗∗^/0.63^∗∗∗^	0.28^∗∗∗^/0.52^∗∗∗^	0.83/0.96
*M*	1.04/1.96	2.36/2.25	1.76/0.98
Variance	0.84/0.92	0.83/0.89	0.65/0.91


*Diagonal: reliability coefficients. For identification of the mean structure, the first threshold of each factor has been fixed to 0. ^∗∗∗^*p* < 0.001.*

### Convergent and Discriminant Validity

The CT-C(M–1) model fit the data well, χ^2^_WLSMV_ (237, *N* = 329) = 304.23, *p* < 0.001, CFI = 0.992, TLI = 0.991, RMSEA = 0.032, *p*_RMSEA_ > 0.999 (Table 2, Model 2a). The standardized estimates of thresholds, factor loadings, and error variances of the items are presented in Table 4. The loadings of the non-reference method indicators on the trait factors of the reference method are correlations between true teacher reports and true self-reports, and they show the degree of convergent validity between the teacher reports and the self-reports at the item level. The trait factor loadings were all significant and ranged from 0.14 (Tpiq 8) to 0.63 (Tpiq 9). This indicated that the convergent validity between self-ratings and teacher ratings differed considerably. The convergent validities were highest for the academic self-concept (0.58 ≤ λ ≤ 0.63) and lowest for social inclusion (0.14 ≤ λ ≤ 0.32). The standardized method factor loadings show the degree of method-specificity of the non-reference method (teacher ratings). The method factor loadings were high (0.66 ≤ λ ≤ 0.90), indicating a rather high level of method specificity for the teacher reports.

**Table 4:** Standardized estimates of thresholds (τ), factor loadings (λ), error variances [Var(ε)], and variance components in the CT-C(M–1) model.

	Threshold			TF		MF			VC		
					
	τ_1_	τ_2_	τ_3_	λ	*SE*	λ	*SE*	*Var*(𝜀)	*CO*	*MS*	*Rel*
**Emotional inclusion**											
Spiq1	-1.12	-0.22	0.90	0.90	0.02	–	–	0.19	0.81		0.81
Spiq4	-1.03	-0.27	0.82	0.71	0.04	–	–	0.50	0.50		0.50
Spiq7	-1.04	-0.35	0.90	0.92	0.02	–	–	0.15	0.85		0.85
Spiq10	-1.03	-0.10	1.09	0.82	0.02	–	–	0.34	0.66		0.66
Tpiq1	-1.87	-0.80	0.72	0.40	0.06	0.85	0.04	0.12	0.16	0.72	0.88
Tpiq4	-1.59	-0.57	0.47	0.36	0.08	0.82	0.03	0.20	0.13	0.67	0.80
Tpiq7	-1.96	-0.79	0.73	0.39	0.06	0.88	0.03	0.08	0.15	0.77	0.92
Tpiq10	-1.82	-0.57	0.89	0.50	0.07	0.81	0.04	0.09	0.25	0.66	0.91
**Social inclusion**											
Spiq2	-2.25	-1.30	0.14	0.72	0.04	–	–	0.48	0.52		0.52
Spiq5	-2.36	-1.52	-0.02	0.86	0.03	–	–	0.25	0.75		0.75
Spiq8	-1.97	-1.57	-0.65	0.60	0.08	–	–	0.64	0.36		0.36
Spiq11	-2.36	-1.48	-0.02	0.89	0.03	–	–	0.21	0.79		0.79
Tpiq2	-1.97	-0.55	0.62	0.21	0.07	0.81	0.04	0.30	0.04	0.65	0.70
Tpiq5	-2.09	-1.12	0.41	0.25	0.08	0.90	0.02	0.13	0.06	0.81	0.87
Tpiq8	-1.72	-1.06	-0.10	0.14	0.06	0.73	0.05	0.44	0.02	0.54	0.56
Tpiq11	-2.25	-1.10	0.42	0.32	0.07	0.89	0.02	0.10	0.10	0.80	0.90
**Academic self-concept**											
Spiq3	-1.57	-0.40	0.74	0.80	0.03	–	–	0.36	0.64		0.64
Spiq6	-1.39	-0.39	1.04	0.67	0.03	–	–	0.55	0.45		0.45
Spiq9	-1.76	-0.66	0.77	0.80	0.03	–	–	0.35	0.65		0.65
Spiq12	-1.87	-1.11	0.38	0.67	0.03	–	–	0.55	0.45		0.45
Tpiq3	-1.13	-0.18	0.96	0.62	0.04	0.73	0.03	0.09	0.39	0.53	0.91
Tpiq6	-1.02	-0.08	0.77	0.58	0.04	0.80	0.03	0.03	0.33	0.63	0.97
Tpiq9	-0.99	-0.17	0.79	0.63	0.04	0.74	0.03	0.06	0.40	0.54	0.94
Tpiq12	-1.22	-0.29	0.47	0.60	0.04	0.66	0.03	0.21	0.36	0.43	0.79


*N = 329. CT-C(M–1), correlated trait-correlated method minus one. Spiq*_i_*, self-report indicator *i*; Tpiq_i_, teacher report indicator *i*; TF, trait factor (self-report); MF, trait-specific method factor (teacher report); VC, variance components; SE, standard error; CO, consistency; MS, method specificity; Rel, reliability.*

Table 4 also shows the estimated variance components at the item level. The reliability, consistency, and method-specificity coefficients were calculated by using formulas provided by Eid et al. (2003). As indicated by the factor loadings, the variance components demonstrated that the method-specificity of the non-reference method (teacher reports) was higher than the consistency between the reference (self-reports) and non-reference method for all items.

In Table 5, the variance components for both observed and true-score variables of the aggregated CT-C(M–1) model are reported. Again, the reliabilities of the scales (especially those of the teacher ratings) were high. The latent correlations between the true score variables of the reference and the non-reference method were computed by taking the square root of the consistency coefficient. They represent correlations between the self-reports and teacher ratings corrected by measurement error (Eid et al., 2003). The consistency coefficients varied considerably across the three scales. The consistency coefficient of the true-score variables of the teacher ratings was 0.07 for social inclusion, 0.19 for emotional inclusion, and 0.41 for academic self-concept. Hence, between 19 and 41% of the teacher ratings could be explained by the self-reports. Latent correlations between self-reports and teacher reports were 0.27 for social inclusion, 0.44 for emotional inclusion, and 0.64 for academic self-concept, indicating low to relatively high convergent validity. In sum, for each scale, the method specificity was larger than the consistency.

**Table 5:** Estimated variance components in the aggregated CT-C(M–1) model.

	Observed variables			True-score variables		
		
Rating	Reliability	Consistency	Method specificity	Consistency	Method specificity	Latent correlation^a^
**Emotional inclusion**						
Self	0.90	0.90		1.00		
Teacher	0.97	0.19	0.78	0.19	0.81	0.44
**Social inclusion**						
Self	0.86	0.86		1.00		
Teacher	0.93	0.07	0.86	0.07	0.93	0.27
**Academic self-concept**						
Self	0.83	0.83		1.00		
Teacher	0.97	0.40	0.57	0.41	0.59	0.64


*CT-C(M–1), correlated trait-correlated method minus one. ^*a*^Latent correlation with the reference method ($\sqrt{consistency}$).*

The correlations of the trait and trait-specific method factors in the CT-C(M–1) model are reported in Table 6. These correlations indicate the discriminant validity at the level of the reference method (self-reports). The correlations between the trait factors were all significant and ranged from 0.28 to 0.50. The correlations indicated that self-ratings of emotional inclusion did not discriminate substantially from the academic self-concept (*r* = 0.50) and social inclusion (*r* = 0.42), whereas the discriminant validity for social inclusion with respect to academic self-concept was better (*r* = 0.28).

**Table 6:** Correlations of the trait and trait-specific method factors in the CT-C(M–1) model.

	Trait factors			Trait-specific method factors		
		
	EMO-S	SOC-S	ASC-S	EMO-T	SOC-T	ASC-T
*Trait factors*						
EMO-S	1.00					
SOC-S	0.42^***^	1.00				
ASC-S	0.50^***^	0.28^***^	1.00			
*Trait-specific method factors*						
EMO-T		-0.02	0.27^***^	1.00		
SOC-T	0.05		0.23^***^	0.70^***^	1.00	
ASC-T	-0.05	-0.10		0.51^***^	0.47^***^	1.00


*Empty cells indicate non-admissible correlations that are fixed to 0. CT-C(M–1), correlated trait-correlated method minus one; S, self-report; T, teacher report; EMO, emotional inclusion, SOC, social inclusion, ASC, academic self-concept. ^∗∗∗^*p* < 00.001.*

The correlations of the trait-specific method factors refer to the generalizability of method effects across traits. When these correlations are high, method effects can be generalized across traits. The correlations between the method factors were all significant and positive, ranging from 0.47 to 0.70. For example, the high correlation between students’ emotional and social inclusion (*r* = 0.70) means that teachers either over- or underestimate both traits in a similar manner. In summary, the positive and relatively high correlations of the trait-specific method factors indicate that teachers’ ratings of students’ inclusion can be generalized to some extent across traits. However, a model with only one single method factor fit the data significantly worse, Δχ^2^_WLSMV_ (2, *N* = 329) = 67.31, *p* < 0.001. This indicated that the method effects are not homogeneous (unidimensional) across traits.

The correlation of the method factor belonging to one trait and the trait factor of another trait indicates the discriminant validity corrected for influences that are due to the reference method. Two of six of these correlations were significant. Students with high self-reported academic self-concept had higher teacher ratings with regard to their emotional (*r* = 0.27) and social (*r* = 0.23) inclusion.

### Measurement Invariance Across Self-Reports and Teacher Reports in the LD Model

Before explaining the method effects, an LD model was applied and examined for measurement invariance. The first of the LD models (Table 2, Model 3a) tested whether the factorial structure was consistent, allowing parameters to be freely estimated across student and teacher ratings. This baseline model showed a good global fit to the data, χ^2^_WLSMV_ (228, *N* = 329) = 304.23, *p* < 0.001, CFI = 0.992, TLI = 0.991, RMSEA = 0.032, *p*_RMSEA_ > 0.999 (Table 2, Model 3a), supporting configural invariance. Weak measurement invariance requires that factor loadings are equal across students’ and teachers’ ratings. The second LD model likewise fitted the data well, χ^2^_WLSMV_ (237, *N* = 329) = 314.22, *p* < 0.001, CFI = 0.992, TLI = 0.991, RMSEA = 0.031, *p*_RMSEA_ > 0.999 (Table 2, Model 3b). The comparison between the first and the second model provided support for weak measurement invariance, which was indicated by a non-significant Δχ^2^ difference value: Δχ^2^(9) = 16.44, *p* = 0.06, and either no change (ΔCFI and ΔTLI = 0.000) or no substantial change (ΔRMSEA = 0.001) in model fit indices. Strong measurement invariance tests whether item intercepts and factor loadings are equal. The fit statistics for this third model were as good as those for the second model: χ^2^_WLSMV_ (258, *N* = 329) = 339.94, *p* < 0.001, CFI = 0.992, TLI = 0.991, RMSEA = 0.031, *p*_RMSEA_ > 0.999 (Table 2, Model 3c). The strong measurement model was rejected by the chi-square difference test, Δχ^2^(21) = 39.32, *p* = 0.01. However, all fit indices were as good as for the weak measurement model (ΔCFI, ΔTLI, and ΔRMSEA = 0.000). Based on these results, we concluded that the assumption of strong measurement invariance across students’ and teachers’ ratings was tenable.

### Effects of Student Characteristics on Self-Reports and General Method Effects

Table 7 shows the correlations, means, and variances in the LD model. The mean of the LD method factor corresponds to the general method effect, which is defined as the difference between the true score variable of the non-reference method and the true score variable of the reference method (Geiser et al., 2012; Koch et al., 2017). The sign of the latent difference method factors indicates over- or underestimation. The significant mean values of the method factors thus suggested that teachers overestimated emotional inclusion compared to students’ self-reports (*M* = 0.37), whereas social inclusion (*M* = -0.47) and academic self-concept (*M* = -0.31) were underestimated.

**Table 7:** Factor means, variances, and correlations in the LD model.

	Trait factors: Self-report			Difference method factors: Teacher – self		
		
	EMO	SOC	ASC	EMO	SOC	ASC
*M*	-	-	-	0.84^**^	-1.08^**^	-0.42^**^
*M_Standardized_*	-	-	-	0.37^**^	-0.47^***^	-0.31^**^
Var	5.58	3.95	1.78	5.10	5.38	1.84
EMO T – S	-0.70^***^	-0.30^***^	0.16	1.00		
SOC T – S	-0.23^**^	-0.64^***^	-0.01	0.55^***^	1.00	
ASC T – S	-0.13	-0.15	-0.16	0.40^***^	0.43^***^	1.00


*LD, latent difference; S, self-report; T, teacher report; T – S, teacher minus self-report. EMO, emotional inclusion, SOC, social inclusion, ASC, academic self-concept. All parameters set to 0 by definition are represented by a dash. ^∗∗^*p* < 0.01, ^∗∗∗^*p* < 0.001.*

The negative significant correlation coefficients between the reference factors for emotional and social inclusion and the latent difference method factors for emotional and social inclusion demonstrated, for example, that the higher the self-reported emotional inclusion was, the smaller the difference between teachers’ reports and students’ self-reports (*r* = -0.70). For academic self-concept, no significant associations between the reference factors and the latent difference method factors were reported.

To explain general method effects, the explanatory variables (gender, age, and SEN) were related to the latent difference method factors in the LD model. The fit statistics for the LD model with explanatory variables (Table 2, Model 3d) suggested a good model fit, χ^2^_WLSMV_ (314, *N* = 329) = 384.82, *p* = 0.004, CFI = 0.993, TLI = 0.992, RMSEA = 0.026, *p*_RMSEA_ > 0.999.

Table 8 shows the estimated standardized regression coefficients of the explanatory variables on trait factors (self-report) and method factors in the LD model. Gender had a small positive effect on the trait factor emotional inclusion (*r* = 0.25), indicating that girls felt better at school than boys. In contrast, girls had a somewhat lower academic self-concept than boys (*r* = -0.12). Students’ age was negatively associated with emotional inclusion (*r* = -0.11). There was no significant effect of SEN on the students’ self-reports. The general method effect regarding academic self-concept can be explained to some extent by gender and SEN: the discrepancy between students’ self-reports and teachers’ ratings of students’ academic self-concept was larger (although to a small extent) for girls compared to boys (*r* = 0.17). In contrast, there was a moderate negative effect for students with SEN (*r* = -0.42). Moreover, the discrepancy between students’ self-reports and teachers’ reports of students’ emotional inclusion was partly due to the SEN status (*r* = -0.13).

**Table 8:** Standardized regression coefficients of gender, age, and SEN on trait and method factors in the LD model.

	Trait factors: Self-report			Difference method factors: Teacher – self		
		
	EMO	SOC	ASC	EMO	SOC	ASC
Gender (female)	0.25^∗∗∗^	0.11	-0.12^∗^	-0.10	-0.01	0.17^∗^
Age	-0.11^∗∗^	0.01	-0.08	-0.04	-0.12	-0.18
SEN (with)	0.08	0.00	-0.08	-0.13^∗^	-0.10	-0.42^∗^


*SEN, special educational needs; LD, latent difference; EMO, emotional inclusion, SOC, social inclusion, ASC, academic self-concept. Coding: SEN 0 = without, 1 = with; Gender 0 = male, 1 = female. ^∗^*p* < 0.05, ^∗∗^*p* < 0.01 ^∗∗∗^*p* < 0.001.*

## Discussion

In this study, we took a closer look at the consistency between the students’ self-reports and teachers’ ratings of students’ emotional and social inclusion at school, as well as their academic self-concept by using the PIQ-S and PIQ-T. To this end, we applied a CT-C(M–1) model, which is an appropriate CFA-MTMM model for the analysis of convergent validity and method effects of structurally different methods (e.g., Eid et al., 2008). Furthermore, an LD model with the explanatory variables gender, age, and SEN was estimated to explain general method effects between students’ self-reports and teachers’ ratings of students’ inclusion at school.

First, the results of the CCFA models provided support for the three-factor structure of the PIQ. Since each scale consists of only four items, the reliability of the scales was relatively high, particularly for the PIQ-T. As expected, ω coefficients were higher than α coefficients. Although measurement errors can thus be considered of minor importance, latent variable modeling approaches are preferred because these not only allow to account for measurement errors, but also to explicitly model sources of variation or unobserved heterogeneity (Muthén, 2002).

Second, the results of the CT-C(M–1) model demonstrated low to moderate convergent validity between students’ self-reports and teachers’ ratings of students’ inclusion at school, indicating that teachers’ reports were in rather weak agreement with the students’ reports. More specifically, convergent validity was higher for students’ academic self-concept than for social inclusion and emotional well-being at school. This is in line with previous studies (Praetorius et al., 2013; Urhahne and Zhu, 2015). Moreover, our study added evidence by using a CFA-MTMM approach to examine consistency and method-specificity of teachers’ reports in contrast to students’ self-reports. Taken together, for all items and scales, the method-specificity was larger than the consistency between students’ self-reports and teachers’ reports. As noted by Yu et al. (2016), method effects tend to be strong for psychological measures, particularly for self-reports of well-being (Eid, 2018). In our study, the high correlations between the trait-specific method factors suggested generalizability of the method effects, meaning that teachers either over- or underestimated students’ emotional and social inclusion and their academic self-concept in a similar manner. Additional analyses, however, indicated that method effects were not homogenous.

Third, the LD model provided further information relating to (general) method effects. Cumulatively, teachers overestimated students’ emotional inclusion and underestimated their academic self-concept and social inclusion. At the same time, the findings suggested that the higher the students’ emotional or social inclusion was, the smaller the discrepancy between the students’ self-reports and teachers’ ratings of both constructs was (and *vice versa)*. The student characteristics included in the LD model could account for the differences only to some extent. Students’ age had no effect on the discrepancy relating to emotional inclusion. Given the small range of students’ age, this is hardly surprising. Age was, however, negatively associated with students’ emotional inclusion, which is in accordance with the decline of positive emotional states across early adolescence (Larson et al., 2002). Gender could explain the general method effect with regard to academic self-concept to a small extent insofar that the discrepancy between students’ self-reports and teachers’ ratings was slightly larger for girls than for boys. This is likely due to the fact that girls have a somewhat lower academic self-concept than boys, whereas teachers tend to rate the self-concept of girls higher than that of boys (Praetorius et al., 2017). SEN seems to have the strongest influence on general method effects (or method bias) relating to the ratings of students’ inclusion. Whereas the effect of SEN was rather small for emotional inclusion, there was a moderate negative effect on the difference between students’ self-reports and teachers’ ratings of students’ academic self-concept. According to labeling theory, arguments suggest that this is due to stigmatization of students with SEN by teachers (e.g., Dunn, 1968). In the literature, it is also argued that students with SEN with regard to learning are less able to appropriately assess their academic competences (Marsh et al., 2006). However, as already indicated, the study of Zurbriggen et al. (2017) found no bias for the items of the PIQ-S in relation to learning difficulties. Against this backdrop, it seems important to pay more attention to possible stigmatization effects or bias in the (external) assessment of subjective characteristics of students with SEN. Teachers should therefore systematically compare their ratings with the students’ self-reports.

The low consistency between students’ self-reports and teachers’ ratings could, however, lead to the conclusion that only one of the two perspectives should be assessed. In research on well-being, self-reports are the most widely used assessment method because the person himself or herself best knows his or her well-being (Eid, 2018). The latter also applies to students with SEN. This underscores the use of self-report as a standard or reference method in the present study. However, self-reports might be biased. In a study by Venetz and Zurbriggen (2016), for example, students evaluated their emotional experience in the classroom more positively in retrospect than *in situ* (i.e., the “rosy view” effect). On the part of teachers, the results suggest a possible halo effect, as teachers tend to rate emotional and social inclusion higher for students with a high academic self-concept.

Nevertheless, our findings highlight the importance of using multiple methods or informants (see also Yu et al., 2016). MTMM designs are favored for several reasons. In their groundbreaking work, Campell and Fiske (1959) already pointed out that a single method may not provide a valid representation of the construct under consideration. Results relying on a single method (or informant) may be specific to that method and, thus, can be limited (Geiser et al., 2010). Consequently, single-method investigations are often less informative than studies combining multiple sources.

We are in complete agreement with Koch et al. (2017) and consider method effects to be an important source of information. In making assessments about students’ inclusion, one should bear in mind that students’ self-reports and teachers’ ratings provide different information in some respects. In (inclusive) education, these inconsistencies could be used as diagnostic information. If a teacher, for example, knows about the comparatively low academic self-concept of a student, he/she understands why the student tends to choose rather simple tasks and can thus help to choose appropriate tasks in the context of personalized teaching. Being aware of the student’s own view may have an impact on the teacher’s behavior.

### Limitations and Future Directions

In the present study several limitations remain and they provide suggestions for future research. First, the sample was composed of Austrian grade 8 students and their teachers. Thus, the findings may not be generalizable to other countries and other grade levels. As in Austrian’s secondary schools, most of the subjects are taught by subject teachers, the teachers observed the students only for a limited number of lessons per week. Hence, the consistency between students’ self-reports and teachers’ ratings might be smaller compared to that of the primary schools in which class teachers are mainly responsible for all subjects. Futures studies could, for example, assess the perspective of different subject teachers in order to gain more insights into the consistency of different teachers’ ratings of students’ emotional and social inclusion. Second, a larger sample size would have been preferable, even though the sample size was acceptable for a CT-C(M–1) model with three traits, two methods, and four indicators. According to Monte Carlo simulations performed by Nussbeck et al. (2006), the WLSMV estimator generally provided adequate results under comparable conditions. However, given the relatively small sample size, we could not apply a CT-C(M–1) model with transformed explanatory variables or latent interaction effects to analyze moderated individual method effects or moderated conditional method effects (Koch et al., 2017). Third, since indicator-specific residual factors were not included in the MTMM models, inhomogeneities among indicators were not captured (Geiser et al., 2014). Finally, in the LD model, the explanatory variables and the method variables were not corrected for confounding influences of the trait factors of the reference method. Thus, the psychometric meaning of the latent method factors differed for the CT-C(M–1) model and the LD model, and this had to be taken into consideration when interpreting the results of both models (Koch et al., 2017).

Despite these limitations, the present study contributes our understanding of teachers’ assessment accuracy by focusing on the consistency between students’ self-reports and teachers’ ratings of students’ emotional and social inclusion and their academic self-concept. Furthermore, the findings add evidence for the psychometric properties of the German PIQ-S and PIQ-T while also demonstrating that the PIQ is a valuable screening instrument for use in both research and applied work. In this vein, future studies could examine the psychometric properties of the English PIQ or those of other language versions.

## Data Availability

The datasets for this study will not be made publicly available due to data protection rights.

## Ethics Statement

The consent obtained from the teachers and the parents of the participants was both written as well as informed. The ethic committee of the regional school board gave the ethical approval for the present study.

## Author Contributions

Lead by the initiative of MV, all authors planned the study, wrote the manuscript, and intensively discussed research questions and the structure of all parts. SS took the lead of the data collection, while MV and CZ conducted the analyses. MV wrote parts of the methods section and contributed mainly to the results. CZ contributed to the methods section and to the results section, was mainly responsible for the discussion and reworked the introduction. SS wrote the sample description, parts of the introduction and of the discussion.

## Conflict of Interest Statement

The authors declare that the research was conducted in the absence of any commercial or financial relationships that could be construed as a potential conflict of interest.

## Footnotes

1 www.piqinfo.ch
